# A novel function of membrane-associated collagen in cancer metastasis

**DOI:** 10.18632/oncotarget.26821

**Published:** 2019-04-05

**Authors:** Hui Zhang, Ren Xu

**Affiliations:** Ren Xu: Markey Cancer Center, University of Kentucky, Lexington, KY, USA

**Keywords:** extracellular matrix, cancer metastasis, type XIII collagen, anoikis, cancer stem cell

Extracellular matrix (ECM) remodeling is necessary for cancer progression and metastasis [1, 2]. Collagen is the major ECM component in tumor tissue. Based on the protein structure and localization, the collagen family can be divided into several groups, including fibrillar collagen, basement membrane collagen, and membrane-associated collagen. Roles of fibrillar collagen and basement membrane collagen in cancer development and progression have been reported in many studies [2-4]. However, little attention has been given to the function of membrane associated collagen, such as collagen XIII, collagen XXIII, and collagen XXV, in cancer progression.

Collagen XIII is a type II transmembrane protein and folds in an opposite fashion to the fibrillar collagens. It has a membrane spanning region near the NC1 domain and a large extracellular region with a short intracellular portion [5]. Collagen XIII plays very important roles in cell-cell interaction at neuromuscular junction-inflammatory reaction and immunomodulation [6]. Although increased collagen XIII expression has been detected in tumor tissue, function of collagen XIII in cancer progression has not been determined until recently. Our study showed that collagen XIII expression is induced in breast cancer tissue compared with normal mammary gland, and that increased mRNA levels of collagen XIII is associated with poor prognosis and cancer metastasis [7]. Silencing collagen XIII expression in metastatic breast cancer cell lines inhibits cancer cell invasion and significantly reduces cancer cell colonization at the secondary organs, indicating that membrane associated collagen XIII contributes to cancer metastasis.

To understand how collagen XIII promotes breast cancer metastasis, we first identified the downstream pathway of collagen XIII in cancer cells. α1β1 integrin has been identified as a potential collagen XIII receptor in CHO cells [8]. We confirmed that collagen XIII expression induces β1 integrin activation in mammary epithelial cells. Treatment with AIIB2, a functional blocking antibody of β1 integrin, suppresses collagen XIII-induced cell migration, invasion, and mammosphere formation. These results suggest collagen XIII promotes cell invasion and mammosphere formation at least partially through the β1 integrin pathway. We also performed co-culture experiments by mixing GFP-labeled collagen XIII-silenced MDA-MB-231 cells with non-labeled wild type MDA-MB-231 cells. Interestingly, wild type MDA-MB-231 cells could not rescue cell invasion in the collagen XIII-silenced cells. These results suggest that collagen XIII promotes cancer cell invasion in a cell-autonomous manner.

**Figure 1:**
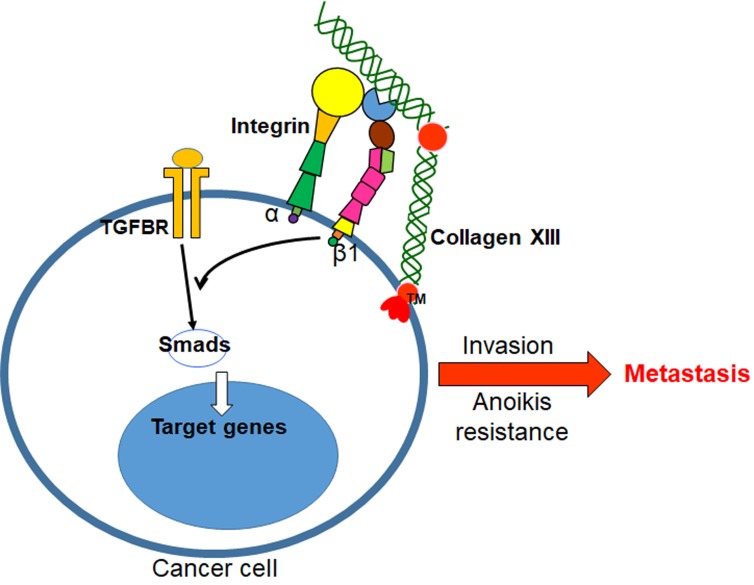
A diagram showing the mechanism by which collagen XIII promotes breast cancer metastasis

β1 integrin plays important roles in the activation of the TGF-β pathway, and abnormal activation of TGF-β signaling promotes cancer progression by inducing EMT and invasion. Results from the TGF-β-induced TPA response elements (TREs)-driven luciferase report assay show that collagen XIII enhances the TGF-β pathway, while AIIB2 treatment inhibits the collagen XIII-induced TGF-β pathway. Therefore, collagen XIII may induce the TGF-β signaling through β1 integrin, and subsequently promotes cancer progression (Figure 1).

Cancer relapses and metastasis depend on the survival, expansion, and differentiation of cancer stem cells. We found that collagen XIII is highly expression in stem cell-enriched cancer cell populations, and that collagen XIII is required for tumorsphere formation. Most epithelial cells die right after they are detached from extracellular matrix, and this kind of cell program death called anoikis. However, cancer stem cells acquire the anoikis resistance, which is crucial for the cancer cell survival during cancer metastasis [9]. We show that increased collagen XIII expression enhances anoikis resistance in cancer stem cells. Importantly, blocking β1 integrin function abolishes collagen XIII-induced anoikis resistance. These results suggest that collagen XIII promotes breast cancer metastasis by enhancing cancer cell stemness and its associated anoikis resistance.

Stromal cells such as cancer-associated fibroblasts are considered as the major source of collagen in tumor tissue; however, recent studies show that cancer cells also produce significant amount of collagen in tumor tissues [10]. We found that the cancer cell-derived collagen XIII enhances cancer cell survival, invasion, and colonization. Therefore, targeting collagen XIII-integrin interaction may be a promising strategy to halt cancer progression.
